# Intercept‐AD: Development and qualification of a highly sensitive immunoassay to detect total sabirnetug (ACU193) in human CSF

**DOI:** 10.1002/alz.094777

**Published:** 2025-01-09

**Authors:** Hao Zhang, Sarah Monsheimer, Jennifer Gillies, Derrick Johnson, Nathan McOwen, Erika N Cline, Mahsan Rafizadeh, Robert A Dean, Jerome A Moore, Jasna Jerecic

**Affiliations:** ^1^ Acumen Pharmaceuticals, Charlottesville, VA USA; ^2^ Meso Scale Diagnostics, LLC, Rockville, MD USA; ^3^ B2S Life Sciences, Indianapolis, IN USA

## Abstract

**Background:**

Sabirnetug (ACU193) is a humanized monoclonal antibody targeting soluble amyloid beta (Aβ) oligomers, which are early contributors to the pathogenesis of Alzheimer’s Disease (AD). An ultrasensitive assay was developed to measure sabirnetug pharmacokinetics (PK) in CSF of individuals with early AD in the Phase 1 study INTERCEPT‐AD (NCT04931459).

**Method:**

The immunoassay was developed on the Meso Scale Diagnostics (MSD) S‐PLEX with improved sensitivity through TURBO‐TAG® technology. Twenty‐four anti‐sabirnetug antibody pairs were screened. Assay selectivity, target interference, dilutional linearity, and stability were evaluated. Assay accuracy and precision were tested, and the lower limit of quantitation (LLOQ) was determined. Target interference was tested by spiking various concentrations of Aβ oligomers and monomers into the sabirnetug calibrators prepared in CSF. Total drug (sabirnetug bound and unbound to AβOs) concentrations were measured in individual study participant CSF samples.

**Result:**

Two anti‐idiotypic antibodies against sabirnetug were selected for capture and detection based on sensitivity and background of blank matrix. Final minimal required dilution (MRD) was 1:4. Optimized concentrations for capture and detection antibodies were 0.0625 µg/mL and 0.2 µg/mL. Assay stability was 12 hours room temperature and 5 freeze/thaw cycles. No interference with recovery of standard curve was observed with Aβ oligomers or monomers. Dilutional linearity data showed accurate dilution up to 1:65,536. Five selectivity CSF samples were spiked with sabirnetug at high, medium, and low doses. Recovery of selectivity samples at all doses met acceptance criteria. Nine of 10 independent accuracy and precision runs met acceptance criteria. LLOQ (6 pg/mL) and upper limit of quantitation (6000 pg/mL) were established through 9 reproducibility runs. All pre‐dose CSF sabirnetug concentrations were <LLOQ. All post‐dose concentrations were measurable in a dose‐dependent manner.

**Conclusion:**

An ultra‐sensitive CSF total sabirnetug immunoassay was successfully developed on the MSD S‐PLEX platform. Assay qualification demonstrated sensitivity, accuracy and precision, selectivity, specificity, dilutional linearity, and stability of the method. Analysis of CSF samples collected during INTERCEPT‐AD showed the method is applicable for quantification of total drug exposure of sabirnetug in clinical trials.

